# Multiple regulatory mechanisms of hepatocyte growth factor expression in malignant cells with a short poly(dA) sequence in the HGF gene promoter

**DOI:** 10.3892/ol.2014.2702

**Published:** 2014-11-11

**Authors:** KAZUKO SAKAI, MASAYUKI TAKEDA, ISAMU OKAMOTO, KAZUHIKO NAKAGAWA, KAZUTO NISHIO

**Affiliations:** 1 Department of Genome Biology, Faculty of Medicine, Kinki University, Osaka-Sayama, Osaka 589-8511, Japan; 2 Department of Medical Oncology, Faculty of Medicine, Kinki University, Osaka-Sayama, Osaka 589-8511, Japan; 3 Center for Clinical and Translational Research, Kyushu University Hospital, Fukoka, Kyushu 812-8581, Japan

**Keywords:** hepatocyte growth factor, poly(dA), deletion, methylation, cancer

## Abstract

Hepatocyte growth factor (HGF) expression is a poor prognostic factor in various types of cancer. Expression levels of HGF have been reported to be regulated by shorter poly(dA) sequences in the promoter region. In the present study, the poly(dA) mononucleotide tract in various types of human cancer cell lines was examined and compared with the HGF expression levels in those cells. Short deoxyadenosine repeat sequences were detected in five of the 55 cell lines used in the present study. The H69, IM95, CCK-81, Sui73 and H28 cells exhibited a truncated poly(dA) sequence in which the number of poly(dA) repeats was reduced by ≥5 bp. Two of the cell lines exhibited high HGF expression, determined by reverse transcription quantitative polymerase chain reaction and enzyme-linked immunosorbent assay. The CCK-81, Sui73 and H28 cells with shorter poly(dA) sequences exhibited low HGF expression. The cause of the suppression of HGF expression in the CCK-81, Sui73 and H28 cells was clarified by two approaches, suppression by methylation and single nucleotide polymorphisms in the *HGF* gene. Exposure to 5-Aza-dC, an inhibitor of DNA methyltransferase 1, induced an increased expression of HGF in the CCK-81 cells, but not in the other cells. Single-nucleotide polymorphism (SNP) rs72525097 in intron 1 was detected in the Sui73 and H28 cells. Taken together, it was found that the defect of poly(dA) in the *HGF* promoter was present in various types of cancer, including lung, stomach, colorectal, pancreas and mesothelioma. The present study proposes the negative regulation mechanisms by methylation and SNP in intron 1 of *HGF* for HGF expression in cancer cells with short poly(dA).

## Introduction

Hepatocyte growth factor (HGF) is a widely-expressed multifunctional growth and angiogenic factor (1). The activity of HGF is mediated by binding to its receptor, a tyrosine kinase MET, and HGF transduces multiple biological effects in target cells, including adhesion, motility, growth, survival and morphogenesis (2).

The HGF level is frequently increased in advanced cancer patients. HGF is a poor prognostic factor of patients with various types of cancer, including hepatocellular carcinoma and breast cancer (3–5). In addition, HGF is reported to be associated with resistance to molecular target drugs, including EGFR-specific tyrosine kinase inhibitors, in lung cancer (6,7). HGF is secreted by tumor cells, vascular smooth muscle cells, pericytes and fibroblasts. The *HGF* gene promoter in humans and mice has been structurally and functionally analyzed (8–10). Inflammatory cytokines, including interleukin-1- and interleukin-6-responsive elements, are present in the human *HGF* gene (8), which is activated transcriptionally by these cytokines. However, little is known about genomic alteration associated with the expression of HGF. Ma *et al* reported that high expression of HGF is regulated by a short deletion in the poly(dA) repeat sequence in the *HGF* promoter region in breast cancer cells (11). The present study investigated the association between the expression levels of HGF in those cells and additional regulation mechanisms of HGF expression.

## Materials and methods

### Cells and cell culture

Human non-small cell lung cancer (NSCLC) PC-6, PC-7, PC-9 and PC-14 cell lines were provided by Tokyo Medical University (Tokyo, Japan) (12,13). The NSCLC SBC-3 cell line was provided by Okayama University School of Medicine (Okayama, Japan) (13). The NSCLC N231, LK-2, Ma-1 and 11_18 cell lines were provided by the National Cancer Research Institute (Tokyo, Japan) (14–17). The NSCLC A549, H1299, H69, Calu-1, Calu-6, H292, H358, H441, H460, H2087, H1650, H1838, H1975 and HCC827 cell lines were obtained from the American Type Culture Collection (Mannassas, VA, USA). The NSCLC EBC-1 cell line was obtained from the Japanese Collection of Research Bioresources Cell Bank (Osaka, Japan). Human gastric cancer HSC38, HSC43 and HSC58 cell lines were provided by the National Cancer Center Research Institute (18,19). Human gastric cancer cell line, OKAJIMA, was provided by Osaka City University (Osaka, Japan). Human gastric cancer IM95, MKN1, MKN7 and MKN74 cell lines were obtained from the Japanese Collection of Research Bioresources Cell Bank. Human gastric cancer N87 and SNU16 cell lines and pancreatic cancer MIAPaCa cell lines were obtained from the American Type Culture Collection. Human pancreatic cancer Sui87, Sui68, Sui70 and Sui73 cell lines were provided by the National Cancer Center Research Institute (20). Human colon cancer HCC56, SW837, CCK-81, Colo201, Colo320 and WiDr cell lines and human hepatocellular cancer HLE, HLF and Huh7 cell lines were obtained from the Japanese Collection of Research Bioresources Cell Bank. Human hepatocellular cancer HepG2, human breast cancer MDAMB-468 and BT-549 cell lines, the human glioma U251 cell line, the human prostate PC-3 cancer cell line and human mesothelioma H28 and MSTO cell lines were obtained from the American Type Culture Collection. The cell lines were maintained in RPMI-1640 medium supplemented with 10% heat-inactivated fetal bovine serum (FBS; Equitech-Bio, Inc., Kerrville, TX, USA). All cell lines were maintained in a 5% CO_2_, humidified atmosphere at 37°C.

### Sample preparation

Total RNA was extracted from cells using ISOGEN (Nippon Gene Co., Ltd., Tokyo, Japan), according to the manufacturer’s instructions. The cDNA templates were synthesized from 1 μg of total RNA using the GeneAmp^®^ RNA polymerase chain reaction (PCR) kit (Applied Biosystems, Foster City, CA, USA).

DNA was extracted from cells using the QIAamp DNA mini kit (Qiagen, Valencia, CA, USA), according to the manufacturer’s instructions. For the 10 mM stock solution, 5-Aza-2′-deoxycytidine (5-Aza-dC; Sigma-Aldrich, St. Louis, MO, USA) was dissolved in dimethylsulfoxide. Aliquots were prepared and frozen at −80°C. The cells were treated with 5-Aza-dC for 48 h prior to the cells being collected and total RNA extracted.

### Reverse transcription-quantitative PCR (RT-qPCR)

The methods used in the present section have been previously described (21). Briefly, RT-qPCR was performed by using a Premix Ex Taq and Smart Cycler system (Takara Bio, Inc., Shiga, Japan), according to the manufacturer’s instructions. The primers used for RT-qPCR were purchased from Takara Bio, Inc. and were as follows: Forward, 5′-GTAAATGGGATTCCAACACGAACAA-3′ and reverse, 5′-TGTCGTGCAGTAAGAACCCAACTC-3′ for *HGF*; forward, 5′-GCACCGTCAAGGCTGAGAAC-3′ and reverse, 5′-ATGGTGGTGAAGACGCCAGT-3′ for glyceraldehyde-3-phosphate dehydrogenase (*GAPDH*). The cDNA was then used as the template for the qPCR reaction. The PCR conditions were as follows: one cycle at 95°C for 5 min, followed by 40 cycles at 95°C for 10 sec and 60°C for 30 sec. The threshold cycle (Ct) values were determined using Thermal Cycler Dice Real Time System (Takara Bio, Inc.). The experiment was independently performed in triplicate using *GAPDH* as a reference to normalize the data.

### Enzyme-linked immunosorbent assay (ELISA)

The cells were seeded at a density of 2×10^6^ cells per 10-cm dish in medium supplemented with 10% FBS and were cultured for 24 h. The medium was then changed to serum-free medium. Following 48 h of incubation, the conditioned medium was collected to measure HGF production.

HGF concentrations in the cultured medium were determined using a Human HGF Quantikine ELISA kit (R&D Systems, Inc., Minneapolis, MN, USA) according to the manufacturer’s instructions. The absorbance of the samples at 450 nm was measured using VERSAmax (Molecular Devices Japan K.K, Tokyo, Japan). Duplicate examinations of 50 μl of the cell-conditioned medium were performed.

### DNA amplification and fragment sizing

DNA amplification was performed with Ex Taq polymerase (Takara Bio, Inc.). The cycling program was one cycle of 98°C for 1 min and then 30 cycles of 98°C for 10 sec, 60°C for 30 sec and 72°C for 10 sec, followed by one cycle of 72°C for 2 min. PCR fragments of 88 bp were analyzed using the Agilent 2100 bioanalyzer (Agilent Technologies, Palo Alto, CA, USA). The primers used for PCR amplification were as follows, forward, 5′-GGTAAATGTGTGGTATTTCGTGAG-3′ and reverse, 5′-GCTGCCTGCTCTGAGCCCAT-3′.

### Sequencing analysis

DNA sequencing was performed directly on purified PCR products using the BigDye terminator v 3.1 sequencing kit (Applied Biosystems). DNA amplification was performed with Ex Taq polymerase (Takara Bio, Inc.). The cycling program was one cycle at 98°C for 1 min and then 30 cycles at 98°C for 10 sec, 60°C for 30 sec and 72°C for 1 min, followed by one cycle at 72°C for 2 min. Following PCR, the product was purified using the QIAquick PCR purification kit (Qiagen), and then sequenced using the ABI BigDye 3.1 dye terminator V3.1 kit (Applied Biosystems) on an ABI Prism^®^ 3100 DNA Analyzer automated sequencer (Applied Biosystems). The primer and probe sequences used were as follows: Forward primer, 5′-TGTGATTCTTCTCCTCGTGGGGT-3′, reverse primer, 5′-AGCCTGACCGTGACCCTGAA-3′ and sequencing primer, 5′-AGCCTGACCGTGACCCTGAA-3′, for rs11763015 and rs78601897; forward primer, 5′-TGTGATTCTTCTCCTCGTGGGGT-3′, reverse primer, 5′-CCAAGAAACAGTCATTGTCCATAGCCTGTCCC-3′ and sequencing primer, 5′-CCTGGGGACACCAGACAGAGGCTG-3′, for rs3735520 and rs3735521; forward primer, 5′-GCATATTCAGTACTCACGAATTCAA-3′, reverse primer, 5′-TGGGACGGGGCTTGGGTTGGA-3′ and sequencing primer, 5′-CCAGGCATCTCCTCCAGAGGGATCCG-3′, for rs72525097.

## Results

### Sequencing of the poly(dA) repeat in the HGF promoter region

**Figure 1:**
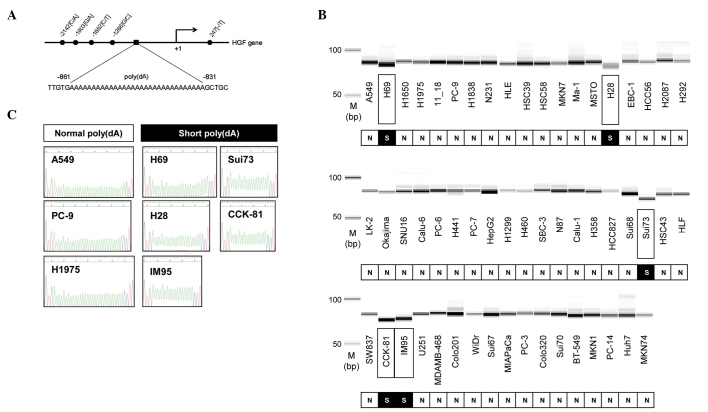
(A) Schematic representation of the human hepatocyte growth factor promoter. The square (■) and circle (●) show the location of poly(dA) and single-nucleotide polymorphisms, respectively. **(**B**)** Capillary electrophoresis of reverse transcription-polymerase chain reaction products derived from 55 cell lines. The product from the A549 cell line [normal poly(dA)] was applied for each assay as a control. DNA size standards (100 and 50 bp) are shown on the left. Truncated fragments were detected in the H69, IM95, CCK-81, Sui73 and H28 cell lines. **(**C**)** DNA sequencing of the poly(dA) region in the eight cell lines. Sequencing analysis revealed the truncated fragment of the poly(dA) sequence in the H69, IM95, CCK-81, Sui73 and H28 cell lines. The dominant peak represents the true fragment length. The right end peaks likely represent polymerase slippage.

The HGF promoter region contains a poly(dA) repeat at ~800 bp upstream from the translation initiation site (Fig. 1A). Based on a previous study (11), the poly(dA) lengths were analyzed by fragment sizing in 55 human cancer cell lines. Shorter fragments were detected in the Sui73, CCK81, IM95, H69 and H28 cells, but not in the remaining cell lines (Fig. 1B). To confirm the fragment size, the poly(dA) region of eight cell lines was sequenced (Fig. 1C). The number of mononucleotide repeats in these cell lines matched with the result of the fragment analysis.

### HGF expression in human cancer cells

**Figure 2:**
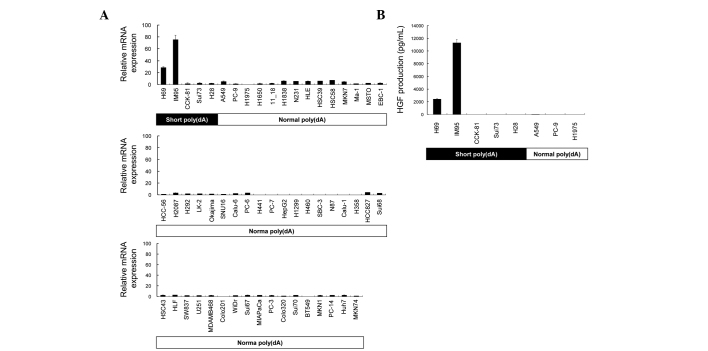
(A) Expression of *HGF* mRNA in human cancer cell lines. cDNA (4 ng RNA/μl; 1 μl) was used for the quantitative polymerase chain reaction. Relative *HGF* mRNA in the each cell lines were normalized by glyceraldehyde-3-phosphate dehydrogenase mRNA levels. The experiment was performed in triplicate, and repeated three times independently. The data are expressed as the mean ± standard deviation of triplicate samples. (B) Production of HGF protein by the eight cell lines. Each cell line was cultured in the medium. HGF concentration in the conditioned medium was determined by enzyme-linked immunosorbent assay. The experiment was performed in triplicate. HGF, hepatocyte growth factor

The levels of *HGF* expression in 55 cell lines were then examined by RT-qPCR (Fig. 2A). RT-qPCR analysis revealed a low expression in the majority of the cell lines. On the other hand, the H69 and IM95 cells expressed high levels of *HGF* mRNA. The expression of HGF mRNA in the H69 and IM95 cells was >12.3- and >32.4-fold higher compared with the average of any cell line other than H69 or IM95, respectively. HGF protein secretion was examined in the eight cell lines (Fig. 2B). The HGF protein was highly secreted by H69 and IM95 cells in the conditioned medium, which was consistent with mRNA expression.

The pattern of poly(dA) length (Fig 1B) and *HGF* expression (Fig. 2A) was compared in 55 cell lines. The expression level of *HGF* was low in all the cell lines with a normal poly(dA) length in the *HGF* promoter region. By contrast, *HGF* expression in the five cell lines with short poly(dA) length in the *HGF* promoter region differed in pattern. High expression of *HGF* was observed in the H69 and IM95, but not in the CCK-81, Sui73, and H28 cell lines. These results suggest that the cell lines with a normal poly(dA) promoter express low levels of *HGF*, and all cell lines with a short poly(dA) do not express high levels of *HGF*. It was hypothesized that the *HGF* expression was suppressed in the CCK-81, Sui73 and H28 cells with short poly(dA) by other mechanisms.

### Effect of 5-Az-dC on HGF expression

**Figure 3:**
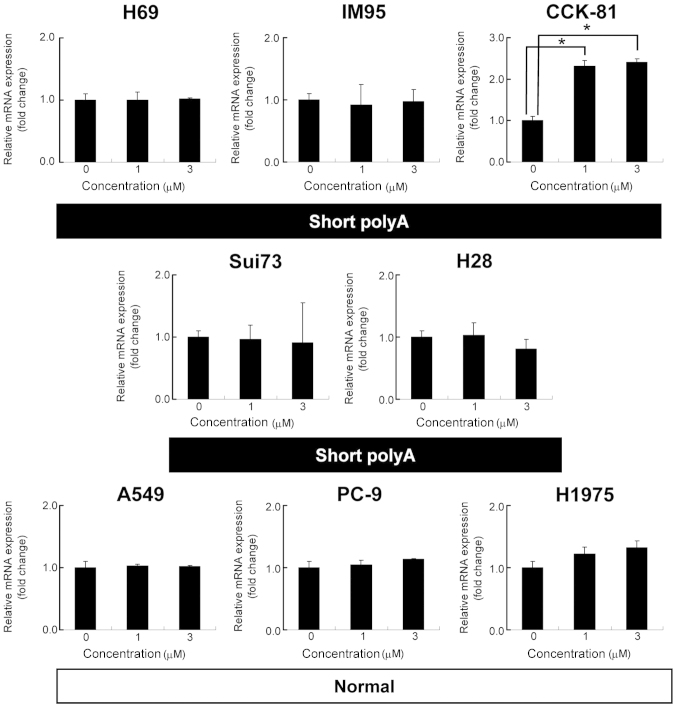
Induction of *HGF* mRNA expression by 5Aza-dC treatment in eight cell lines. The indicated cell lines were treated with 1 or 3 μM 5Aza-dC for 48 h, and total RNA was prepared. Levels of *HGF* and *GAPDH* mRNA were quantified by reverse transcription-quantitative polymerase chain reaction. Relative HGF mRNA was normalized by glyceraldehyde-3-phosphate dehydrogenase mRNA levels. The experiment was performed in triplicate, and repeated three times independently. The data are expressed as the mean ± standard deviation of triplicate samples. ^*^P<0.01 by Student’s t-test. HGF, hepatocyte growth factor.

The present study explored the cause of *HGF* gene suppression in certain cell lines with shorter poly(dA) sequences. It was hypothesized that DNA methylation may silence *HGF* expression in these cell lines. The change in the *HGF* mRNA expression in three cell lines was examined following treatment with 1 or 3 μM 5-Aza-dC for 48 h (Fig. 3). A 2.3- and 2.4-fold increase in *HGF* mRNA was observed in CCK-81 cells when treated with 1 and 3 μM 5-Aza-dC, respectively (P<0.01). No significant change was observed in the Sui73 and H28 cells. These results suggest that DNA methylation may contribute to the silencing of the *HGF* gene in the CCK-81 cells.

### Genotyping of SNPs in the HGF gene

All increased expression of *HGF* in the Sui73 and H28 cells was due to DNA methylation. Next, it was examined whether a specific polymorphism is observed in these cells. The *HGF* genotypes were analyzed upstream of the transcript start (−2200 bp) to intron1 (+300 bp) of 12 cell lines with short poly(dA) (H69, IM95, CCK-81, Sui73, and H28 cells) or normal poly(dA) (A549, PC-9, H1975, HCC827, H1650, 11_18, and Ma-1 cells) by direct sequencing (Table I). The -1903G and -1268G SNPs were detected in all 12 cell lines. The -2142C/A SNP was detected in only the IM95 cell line with short poly(dA). The -1652C/T SNP was detected in four cell lines with shorter and normal poly(dA). The +247T SNP in intron 1 was detected in only the Sui73 and H28 cells with a short poly(dA). These results suggest that insertion of a single T nucleotide at position 247 may be associated with the expression of HGF in the Sui73 and H28 cells with a short poly(dA).

## Discussion

HGF has been identified as a natural ligand of the MET receptor and belongs to the plasminogen family (1). In cancer cells, activation of the MET receptor increases invasion and metastasis, and allows the survival of cancer cells in the bloodstream in the absence of anchorage (2).

In the present study, the deletion mutation on the HGF promoter region was detected in lung, colon, stomach, pancreatic and mesothelial cancer cell lines. The cell lines with short poly(dA) express high levels of *HGF*, whereas all the cell lines with normal poly(dA) do not. This result is consistent with a previous study (11) and short poly(dA) in the *HGF* promoter region has been detected in various types of cancer cell lines and breast cancer. H69 [poly(dA), 24 bp] and IM95 [poly(dA), 17 bp] highly express *HGF*. When comparing these two cell lines, IM95 exhibits a higher *HGF* expression compared with H69 (2.6- and 4.6-fold higher at the mRNA and protein levels, respectively). This is also consistent with the study by Ma *et al*, suggesting that the cells with shorter poly(dA) exhibit higher *HGF* (11).

By contrast, certain cell lines exhibited a short poly(dA) and low *HGF* expression. We considered that the low *HGF* expression in these cell lines may be explained by the methylation or polymorphisms of the *HGF* promoter. There has been no previous paper reporting epigenetic regulation of HGF expression. Recently, DNA methylation analysis of *HGF* promoters was reported. Analysis of bisulfite DNA from BNL 1ME A.7R.1 cells indicated that *HGF* has 35% of CpGs methylated at two sites (22). This finding is considered to be important for elucidating the detailed mechanisms of epigenetic regulation of HGF expression, in order to develop epigenetic therapy for HGF-related cancers.

An increase (by 2.4-fold) in *HGF* expression was induced by 5Aza-dC in Sui73 and H28 cell lines, suggesting that other mechanisms such as acetylation and transcription factors may influence the full expression of HGF.

T insertion in intron 1 (247T) was detected in two cell lines with short poly(dA). It is well-known that polymorphisms in the promoter, but not in introns, influence the transcription of a gene. However, Onouchi *et al* reported that an SNP in intron 1 of 1,4,5-trisphosphate 3-kinase C influenced the transcription of this gene (23). Yamada *et al* reported that polymorphism in intron 1 of transcription factor *EGR3* regulates transcription of this gene (24). Subsequently, it was hypothesized that 247(−/T) may influence the transcriptional levels of HGF in these cell lines, although functional analysis is necessary in future studies. Another SNP was detected in 4/12 cell lines (−1652C/T); however, the influence of this SNP on HGF expression remains unknown.

HGF is an important mediator of epithelial-mesenchymal transition (EMT) (25). Fibroblastic changes in the cells were often observed during EMT induced by HGF and other ligands. The association between SNPs in HGF and EMT may be investigated in future studies.

In conclusion, the present study found that the deletion polymorphism of poly(dA) in the *HGF* promoter was present in various cancer cell lines, including lung, stomach, colorectal and pancreatic cancer, and mesothelioma cell lines. Future experiments with functional analysis of intron 1 polymorphisms may provide a novel negative regulatory mechanism of *HGF* expression.

## Figures and Tables

**Table I:** A list of single nucleotide polymorphisms detected in the 12 cell lines. The poly(dA) length represents the number of deoxyadenosine repeat sequences in the *HGF* gene from the results of direct sequencing. HGF production represents the results of the enzyme-linked immunosorbent assay.

Cell line	poly(dA) length	HGF production (pg/ml)	rs11763015-2142C/A	rs78601897-1903G/A	rs3735520-1652C/T	rs3735521-1268G/C	rs72525097 247(−/T)
H69	24	2443.0	C	G	C	G	-
IM95	17	11297.6	C/A	G	T	G	-
Sui73	17	3.6	C	G	C/T	G	T
CCK-81	16	10.0	C	G	C	G	-
H28	20	6.2	C	G	C/T	G	T
A549	27	26.2	C	G	C	G	-
PC-9	29	3.1	C	G	C	G	-
H1975	28	14.4	C	G	C	G	-
HCC827	28	NT	C	G	C/T	G	-
H1650	29	NT	C	G	C	G	-
11_18	29	NT	C	G	C/T	G	-
Ma-1	28	NT	C	G	C	G	-

NT, not tested; HGF, hepatocyte growth factor.
